# Comparing a Novel Anti-BCMA NanoCAR with a Conventional ScFv-Based CAR for the Treatment of Multiple Myeloma

**DOI:** 10.3390/cells14241944

**Published:** 2025-12-08

**Authors:** Mégane Jassin, Chloé Onkelinx, Valentina Bocuzzi, Bianca E Silva, Oswin Kwan, Alix Block, Sophie Dubois, Coline Daulne, Guillaume Marcion, Sandra Ormenese, Emmanuel Di Valentin, Frédéric Baron, Céline Grégoire, Grégory Ehx, Tham Thi Nguyen, Jo Caers

**Affiliations:** 1 Laboratory of Hematology, GIGA Institute, University of Liège, 4000 Liège, Belgium; 2 Department of Clinical Hematology, University Hospital Center (CHU) of Liège, 4000 Liège, Belgium; 3 Cell Imaging & Flow Cytometry Platform, GIGA Institute, University of Liège, 4000 Liège, Belgium; 4 Viral Vectors Platform, GIGA Institute, University of Liège, 4000 Liège, Belgium; 5 Walloon Excellence in Life Sciences and Biotechnology (WELBIO) Department, WEL Research Institute, 1300 Wavre, Belgium

**Keywords:** MM, BCMA, nanobody, CAR-T cells, immunotherapy

## Abstract

**Highlights:**

**What are the main findings?**

- A novel Nb17-based BCMA nanoCAR-T was successfully engineered and displayed in vitro cytotoxicity, cytokine release, and transcriptomic profiles comparable to the clinically validated scFv-CAR CT103a.
- Nb17-nanoCAR-T showed potent antitumor activity in vivo, fully eradicating myeloma tumors similarly to CT103a.

**What are the implications of the main findings?**

- Single-domain antibody-based CARs can achieve efficacy equivalent to conventional scFv-CAR-T, supporting their use as viable therapeutic alternatives in multiple myeloma.
- The Nb17-nanoCAR-T design highlights the potential of VHH-based CARs for future development of more stable, compact, and potentially less immunogenic cell therapies.

**Abstract:**

Multiple myeloma (MM) is an incurable hematologic malignancy arising from clonal plasma cells, with poor long-term outcomes due to inevitable relapse after conventional therapies. Chimeric antigen receptor (CAR) T-cell immunotherapy targeting B-cell maturation antigen (BCMA) has shown remarkable efficacy in relapsed patients. Conventional CARs employ single-chain variable fragments (scFvs), whereas single-domain antibodies (sdAb or VHHs) offer advantages such as small size, high stability, and potentially reduced immunogenicity. We designed and evaluated a novel anti-BCMA nanoCAR-T based on the VHH Nb17, compared with the conventional scFv-based CAR-T CT103a. Nb17 demonstrated strong BCMA binding and was incorporated into a CAR construct. Both nanoCAR-T and CT103a were generated via lentiviral transduction of primary T cells. Their cytotoxicity, cytokine secretion, degranulation, memory phenotype, and gene expression were assessed in vitro, along with antitumor activity in vivo. Nb17-nanoCAR-T demonstrated specific cytotoxicity, cytokine release (IL-2, TFNa, IFNg), and CD107a degranulation comparable to CT103a. Transcriptomic analysis revealed overlapping pathways between both CARs. Upon rechallenge, both CARs showed enhanced proliferation compared with untransduced T cells. In vivo, Nb17-nanoCAR-T and CT103a eradicated tumors in NSG mice. These findings demonstrate Nb17-nanoCAR-T exhibits potent anti-myeloma efficacy comparable to scFv-based CAR-T, supporting its potential as a promising therapeutic alternative.

## 1. Introduction

Multiple myeloma (MM) is a hematologic malignancy characterized by the accumulation and proliferation of clonal plasma cells in the bone marrow (BM). Although this cancer remains incurable, patient survival has improved substantially with the advent of proteasome inhibitors, immunomodulatory drugs, and, more recently, novel immunotherapeutic approaches [1]. B-cell maturation antigen (BCMA) is highly expressed on the surface of MM cells [2]. New therapeutic strategies, such as bispecific T-cell engagers and chimeric antigen receptor (CAR)-T cells, target this membrane protein [3,4]. CAR-T cells are genetically modified T cells that express chimeric receptors capable of recognizing tumor-associated antigens independently of MHC presentation [5,6]. In 2017, the US Food and Drug Administration (FDA) approved the first CAR-T therapy for B-cell malignancies [5,6,7]. In March 2021, the first anti-BCMA CAR-T, idecabtagene vicleucel, was FDA-approved for MM treatment [8]. In February 2022, CT103a, an anti-BCMA CAR-T immunotherapy, was reported to demonstrate efficacy for MM treatment [9,10,11]. CT103a is a fully human CAR incorporating a single-chain variable fragment (ScFv) derived from a phage display library as its extracellular antigen-recognition domain [12]. At present, the clinical trials with CT103a show an excellent tumor control profile [13].

CAR-T cells can be generated using various approaches, including retroviral or lentiviral transduction, CRISPR-Cas9 gene editing, lipid nanoparticles, or non-viral transposon-mediated methods. The conventional CAR-T cell manufacturing process involves T-cell activation and CAR transduction followed by in vitro expansion prior to infusion. This approach aims to optimize the balance between CAR-T cell yields and their therapeutic potency. Cells are often activated using anti-CD3/CD28 stimulation and are grown in the presence of interleukin-2 (IL-2) or IL-7 and IL-15, allowing for efficient transduction and expansion [14,15,16].

Camelid heavy chain variable domains (VHH, also referred to as nanobodies) are antibody fragments consisting of a monomeric variable domain (sdAb). In comparison to conventional antibodies, the single antigen-binding domain of sdAbs confers a higher stability with a similar specificity. Their small size, robust structure, and enhanced stability make them valuable for therapeutic applications, diagnostic purposes [17,18], or as theragnostic agents when radiolabeled [19]. In 2022, the FDA approved a nanoCAR-T therapy, Ciltacabtagene autoleucel (Cilta-cel), whose antigen-binding domain consists of two VHHs capable of binding two distinct BCMA epitopes [20,21,22].

The purpose of this research is to evaluate how replacing a conventional scFv with a VHH impacts CAR-T cell properties. To do so, we compare a newly generated nanoCAR-T with a standard CAR-T CT103a. Specifically, we aim to assess differences in transduction efficiency, antigen engagement, early activation dynamics, transcriptional profiles, and cytotoxicity upon contact with MM cells both in vitro and in vivo. By defining these functional and molecular properties, this work seeks to provide insight that may inform the optimization of next-generation CAR-T therapies for MM.

## 2. Materials and Methods

### 2.1. Cell Lines and Primary T Cells

LP-1 (Uppsala University, Uppsala, Sweden), RPMI-8226 (Wilhelminen Cancer Research Institute, Wilhelminenspital, Vienna, Austria), MM1.S (Wilhelminen Cancer Research Institute, Wilhelminenspital, Vienna, Austria), MOLP-2 (DSMZ, Braunschweig, Germany), KMS-12-BM (DSMZ, Braunschweig, Germany), and K562 (ATCC, Manassas, VA, USA) cells were cultured at Roswell Park Memorial Institute (RPMI 1640) (Lonza, Verviers, Belgium) supplemented with 10% fetal bovine serum (FBS; Sigma-Aldrich, St-Louis, MO, USA), 2 mM L-glutamine (Lonza, Verviers, Belgium), and 100 U/mL penicillin/streptomycin (P/S; Lonza, Verviers, Belgium). Primary T cells were cultured in CTSTM OpTmizerTM T-cell expansion SFM (Thermo Fisher Scientific, Waltham, MA, USA) supplemented with OpTmizerTM T-cell Expansion Supplement (Thermo Fisher Scientific, Waltham, MA, USA), 2 mM L-glutamine (Lonza, Verviers, Belgium), and 100 U/mL penicillin–streptomycin (P/S; Lonza, Verviers, Belgium) and 5 ng/mL of IL-2 (Peprotech, Neuilly-sur-Seine, France). All cells were cultured at 37 °C in 5% CO_2_.

### 2.2. PBMCs and T Cells Isolation

Human peripheral blood mononuclear cells (PBMCs) were obtained from buffy coats from a registered biobank (Croix-Rouge de Belgique, Liège, Belgium) from healthy adult anonymous donors who gave written informed consent. PBMCs were isolated by Ficoll-Paque density centrifugation (GE Healthcare, Freiburg, Germany). Primary T cells were purified using the EasySep Human T-Cell Isolation Kit (Stemcell Technologies, Vancouver, BC, Canada) according to the manufacturer’s protocol.

### 2.3. Lentiviral Vector Production and Cell Transduction

DNA sequences of both the ScFv and VHH antigen-binders were obtained from publicly available sources [23,24]. Lenti-X 293T cells were co-transfected with lentiviral gene transfer plasmids (ScFv-CT103a-hPGK-puro or VHH-nanoCARNb17-hPGK-puro) designed with VectorBuilder (Guangzhou, China) and packaging plasmids (psPAX2 and a VSV-G-encoding plasmid). Lentiviral supernatants were collected 48 h, 72 h, and 96 h post-transfection, filtered, and concentrated 100× by ultracentrifugation. Lentiviral vectors were titrated with qPCR Lentivirus Titration (Titer) Kit (ABM^®^, LV900, Richmond, BC, Canada) and used to transduce T cells to generate CAR-T cells using a multiplicity of infection (MOI) of 10. Transduction efficiency was analyzed by flow cytometry using a V5-AlexaFluor647 antibody (Thermo Fisher Scientific, Waltham, MA, USA). Cancer cell lines were transduced using the same protocol with different lentiviral gene transfer plasmids (CMV-GFP-hPGK-blasti or CMV-GFP-luciferase-hPGK-blasti), and GFP-positive cells were sorted using the Sony MA900 (Sony Biotechnology, San Jose, CA, USA). Sequences of CT103a [23] and Nb17 [24] are listed in Table S1 (Supplementary data).

### 2.4. Flow Cytometry Staining and Analysis

Membrane staining was performed for both in vitro and in vivo studies. Cells were incubated for 60 min at 4 °C with titrated antibody concentrations before analysis on a FACS CantoTMII (BD Biosciences, San Jose, CA, USA), a CytoFLEX (Beckman Coulter, Brea, CA, USA), or an LSRFortessaTM (BD Biosciences, San Jose, CA, USA). The following antibodies were used: anti-human CD3-BV605 (UCHT1, BD Biosciences, CA, USA), anti-human CD45-BV711 (HI30, BD Biosciences, CA, USA), anti-mouse CD45-Pe-Cy5.5 (30-F11, BD Biosciences, CA, USA), anti-human CD4-PerCP-Cy5.5 (RPA-R4, Sony Biotechnology, San Jose, CA, USA), anti-human CD8-PE-Cy7 (HIT8a, Biolegend, San Diego, CA, USA), anti-human CD45RA-BV510 (HI100, BD Biosciences, CA, USA), anti-human CD62L-AlexaFluor700 (DREG-56, Biolegend, San Diego, CA, USA), and anti-human CD27-PE (M-T271, BD Biosciences, San Jose, CA, USA). To discriminate live from dead cells, samples were then incubated for 10 min at 4 °C with a Fixable Viability Dye eFluor™ 780 (1:10,000 dilution; Invitrogen, Thermo Fisher Scientific). For the detection of Nb17-binding, cells were incubated with an anti-VHH antibody (GenScript Biotech, Piscataway, NJ, USA) followed by an anti-rabbit-AlexaFluor647 secondary antibody (Biolegend, San Diego, CA, USA).

All analyses were performed using FlowJoTM software V10.1 (BD Biosciences, Ashland, OR, USA).

### 2.5. Quantification of BCMA or CAR Cell Surface Expressions and Binding Capacity of VHH Nb17

LP-1, RPMI-8226, MM1.S, MOLP-2, and KMS-12-BM cell lines were labeled with a PE-conjugated monoclonal antibody, while primary T and CAR-T cells were labeled with PE-conjugated BCMA soluble protein (ACROBiosystems, Basel, Switzerland). We used the BD QuantibriteTM Beads (BD Biosciences, San Jose, CA, USA) to quantify the BCMA or CAR expression according to the manufacturer’s instructions. This kit contains beads conjugated at four levels of PE intensity, each corresponding to a defined number of PE molecules per bead. Based on the generated standard curve, the number of PE molecules per cell was calculated after labeling with the PE-labeled antibody anti-BCMA-PE (19F2, Biolegend, San Diego, CA, USA) or soluble protein BCMA-PE (AcroBiosystems, Newark, DE, USA).

### 2.6. Cellular Cytotoxicity Assays and CAR-T Phenotyping and Characterization: Flow Cytometry

CAR-T-dependent tumor-cell cytotoxicity was quantified by flow cytometry. GFP transduced target cells were mixed in co-culture with effector CAR-T cells at different effector-to-target ratios (1:3, 1:1, 3:1) in 96-well plates in RPMI 1640 supplemented with 10% FBS, 2 mM L-glutamine, and 100 U/mL penicillin–streptomycin without IL-2 at 37 °C in a humidified atmosphere with 5% CO_2_. After 24 h to 48 h, target cell viability was assessed by counting live cells using Fixable Viability Dye eFluor™ 780. CAR-T cells were phenotyped and characterized by antibody labeling as described above. For the rechallenging assays, co-cultures were supplemented with MM1.S thrice every 48 h with a ratio of 1:1.

### 2.7. CD107a Degranulation Assays: Flow Cytometry

CD107a degranulation assay: Anti-CD107a antibody (anti-human CD107a (H4A3, Biolegend, San Diego, CA, USA) was added directly to the cell culture at the start of stimulation. CAR-T cells were co-cultured with target MM cells at a 1/1 ratio for 6 h at 37 °C in the presence of GolgiStop (eBioscience™ Protein Transport Inhibitor Cocktail, 500× stock diluted 1:500 to 1× final, Thermo Fisher Scientific, Waltham, MA, USA). After 6 h, cells were washed and surface-stained for CD107a and additional surface markers without permeabilization, then analyzed by flow cytometry. This approach captures transient surface exposure of CD107a during degranulation while preventing receptor internalization.

### 2.8. Cellular Cytotoxicity Assays and Cytokine Productions: ELISA

CAR-T-dependent tumor-cell cytokine production was quantified by ELISA. GFP transduced target cells were mixed in co-culture with effector CAR-T cells at a ratio of effector-to-target 1:1 in 96-well plates in RPMI 1640 supplemented with 10% FBS, 2 mM L-glutamine, and 100 U/mL penicillin–streptomycin without IL-2 at 37 °C in a humidified atmosphere with 5% CO_2_. After 24 h, cells were centrifuged, and supernatants were collected to analyze the IL-2, IFNγ, and TNFα productions by ELISA MaxTM Deluxe Set from Biolegend (San Diego, CA, USA) according to the manufacturer’s protocol. Absorbances were read using the Plate Reader Tristar2 S LB942 number 2076 from Berthold Technologies (Bad Wildbad, Germany).

### 2.9. Bulk-RNA Sequencing

RNA-seq samples were collected at three key co-culture timepoints: 0, 4, and 16 h of co-culture of CAR-T or untransduced T cells with MM1.S. After these timepoints, GFP-CD3+CAR+ (CAR-T) and GFP-CD3+CAR- (T cells) were sorted by fluorescence-activated cell sorting (Sony MA900 flow cell sorter) using CD3-BV605 (UCHT1, BD Biosciences, CA, USA) and/or V5-AlexaFluor647 (Thermo Fisher Scientific, Waltham, MA, USA) antibodies. Bulk RNA samples were prepared from sorted cells using the kit RNeasy^®^ Mini Kit (Qiagen, Hilden, Germany) according to the manufacturer’s protocol. Samples were purified after treatment with DNAse I (Qiagen, Hilden, Germany) and further purified using Zymo columns (Zymo Research, CA, USA). Libraries were sequenced using the Illumina Novaseq 6000. Raw sequencing reads were quality-controlled with FastQC (v0.12.1) to remove adapters and low-quality bases. Cleaned reads were pseudo-aligned to the human reference genome (GRCh38.99) using kallisto (v0.51.1). Gene-level counts were generated with R package tximport (v1.34.0 based on Ensembl gene annotations). Downstream analyses were performed in RStudio (R v4.4.2). Normalization and differential expression analyses were conducted using the DESeq2 package (v1.46.0). Genes with adjusted *p*-value (Benjamini–Hochberg FDR) < 0.05 and the absolute value of the fold-change > 1 were considered significantly differentially expressed. Gene set enrichment analysis (GSEA) was performed with fgsea package (v1.32.0) using Hallmark gene sets. Visualization of results was performed with ggplot2 (v3.5.1).

### 2.10. In Vivo NSG Mouse Model

Ethical approval for the in vivo part of the study was obtained from the institutional animal ethical board (Commission d’Ethique Animale Universitaire de Liège). NSG mice received 2-Gγ γ-irradiation on day −1, followed by tail vein injection of 1 × 10^6^ MM1.S-GFP/luc on day 0. Tumor growth was followed using bioluminescence imaging with the Xenogen IVIS 200 with 3 mg/mouse luciferin injection every 7 to 10 days. Then, 16 to 19 days after tumor injection, when the tumor signal was detectable, 2 × 10^6^ CAR-T were injected intravenously in the tails. Mice were monitored for tumor growth and survival for up to 90 days. Additionally, blood samples were collected via the tail vein every 20–30 days after CAR-T injections to analyze CAR-T phenotype and trafficking using flow cytometry. Two independent cohorts were conducted. In cohort 1 (n = 15), mice were distributed into four groups: MM only (n = 3), MM + Mock T cells (n = 4), MM + CT103a (n = 4), and MM + nanoCAR-T cells (n = 4). In cohort 2 (n = 12 mice), mice were distributed into three groups: MM only (n = 2), MM + CT103a (n = 5), and MM + nanoCAR-T cells (n = 5). In each cohort, all T-cell products were derived from a single healthy donor. Mice were monitored daily for clinical signs and weighed three times per week when their clinical score, based on locomotion, hair texture, hair loss, posture, and activity, was ≤4. For animals with a score > 4, individual daily monitoring and recording of clinical parameters were performed, including daily body weight measurement. Euthanasia was carried out if one of the following endpoints was reached: persistent paraplegia, severe and sustained apathy, or a clinical score > 6.

### 2.11. Statistical Analysis

GraphPad Prism 8.0.1. (GraphPad Software, Inc., La Jolla, CA, USA) was used to perform statistical analysis and graph plotting. Comparisons between two datasets were conducted using paired or unpaired Student’s *t*-tests, with parametric or non-parametric versions applied based on the distribution of the data, thanks to the Shapiro–Wilk normality test (e.g., Gaussian, non-Gaussian). Statistical significance was defined as *p* < 0.05 (*) and defined as follows: *p* < 0.01 (**), *p* < 0.001 (***), *p* < 0.0001 (****). *p*-values above 0.05 were considered non-significant (ns).

## 3. Results

### 3.1. Construction of a nanoCAR Sequence Containing VHH Nb17

**Figure 1:**
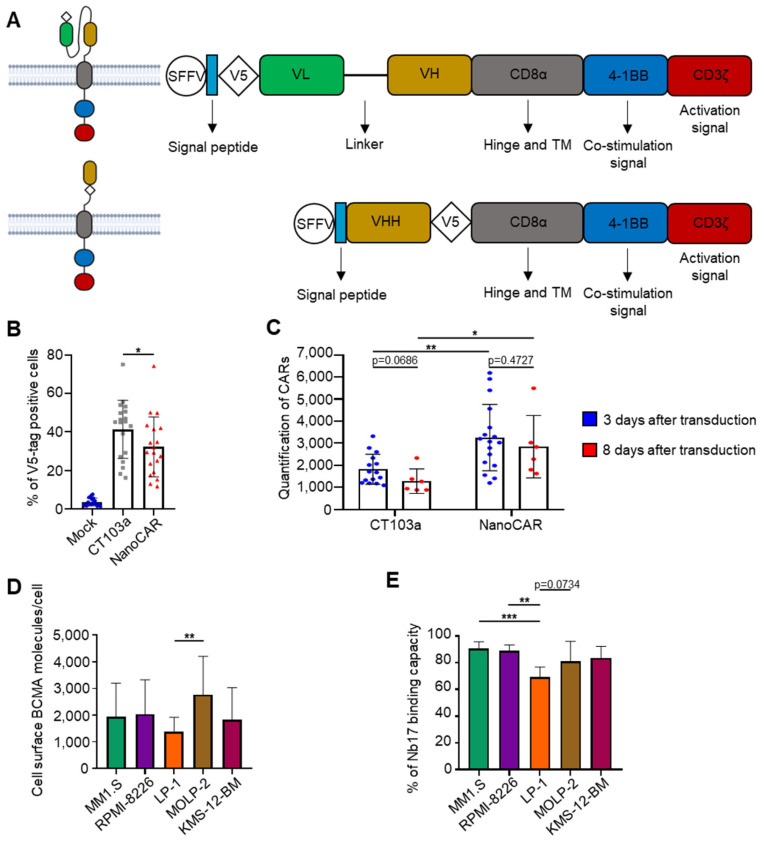
Construction of a nanoCAR sequence containing VHH Nb17. (**A**) Schematic diagram of the second-generation CAR, the FDA-approved scFv-based CAR-T CT103a, and our nanoCAR construct containing VHH Nb17. (**B**) Rate of lentiviral transduction of T cells to generate CT103a and nanoCAR, evaluated using flow cytometry (n = 19 independent donors). (**C**) Analysis of CAR expression stability on the T-cell surface at 3 and 8 days post-transduction (for the condition 3 days after transduction, n = 2 technical replicates for each of 8 independent donors, whereas for the condition 8 days after transduction, 3 of the 8 donors were included, with n = 2 technical replicates per donor). (**D**) Quantification of the number of cell surface BCMA molecules on MM cell lines: MM1.S, RPMI-8226, LP-1, MOLP-2, and KMS-12-BM (n = 14 independent experiments). (**E**) Validation of VHH Nb17 binding to cell-surface BCMA across various MM cell lines (n = 4 independent experiments). Results are presented as the mean value and standard deviation (SD) from several independent experiments. *: *p* < 0.05. **: *p* < 0.01. ***: *p* < 0.001, determined by Mann–Whitney test.

We designed our nanoCAR to be analogous to the scFv-based CAR, CT103a, to evaluate its performance. Both CAR constructs were derived from a second-generation CAR featuring a 4-1BB-based backbone with a CD8 hinge and transmembrane domain. A V5 tag was added to both sequences for detection by flow cytometry. The nanoCAR sequence was composed of SFFVprom-VHH-V5tag-CD8α-(4-1BB)-CD3ζ, while the CT103a sequence was SFFVprom-V5tag-VH-linker-VL-CD8α-(4-1BB)-CD3ζ (Figure 1A). The V5 tag is a short epitope derived from the P and V proteins of the simian parainfluenza virus 5 (SV5). It is widely used for the detection of recombinant proteins because it does not interfere with protein folding or function and can be efficiently recognized by high-affinity monoclonal antibodies. Therefore, the V5 tag has been selected for CAR detection. It was positioned differently in the two constructs to avoid interference with the domain folding; at the N-terminus for the scFv-based CT103a and at the C-terminus for the NanoCAR, as recommended for maintaining antigen-binding integrity of each construct [25,26,27].

Mock T cells, CT103a, and nanoCAR-T-cell types were activated using CD3/CD28 beads and IL-2 for 8 days before being co-cultured with tumor cells. Both CT103a and nanoCAR-T cells were generated through lentiviral transduction at the same MOI (Figure 1B). Interestingly, although nanoCAR-T cells showed lower transduction efficiency than CT103a, the surface density of CAR expression on transduced cells remained equally stable over time. Moreover, nanoCAR-T cells expressed a higher level of CAR per CAR-positive cell, thereby compensating for the lower amount of transduced cells (Figure 1C). A plausible explanation for the higher nanoCAR expression per cell would be the smaller and simpler structure of the VHH domain. As single-domain antibodies, VHHs do not require VH-VL pairing, reducing misfolding, aggregation, and intracellular retention. This may facilitate more efficient CAR processing and trafficking, while the compact extracellular domain likely reduces steric hindrance at the plasma membrane, allowing for a higher density of CAR molecules per cell despite the lower overall transduction rate compared to CT103a. To incorporate the VHH Nb17 sequence for generating our nanoCAR, we assessed its specific binding capacity to cell-surface BCMA. Cell-surface BCMA expression levels were quantified by flow cytometry across various human MM cell lines before incubating them with VHH Nb17. BCMA expression was highest in MOLP-2 (2772 BCMA/cell), followed by RPMI-8226 (2034 BCMA/cell), MM1.S (1942 BCMA/cell), KMS-12-BM (1834 BCMA/cell), and LP-1 (1375 BCMA/cell). K562 did not express BCMA (Figure 1D, Supplementary Data Figure S1). Despite the varying levels of BCMA expression, VHH Nb17 demonstrated specific binding to BCMA (Figure 1E). Thus, we successfully designed a persistent and stable nanoCAR capable of specifically targeting cell-surface BCMA.

### 3.2. In Vitro Efficacy of CT103a and nanoCAR in Killing BCMA+ MM Cell Lines

**Figure 2:**
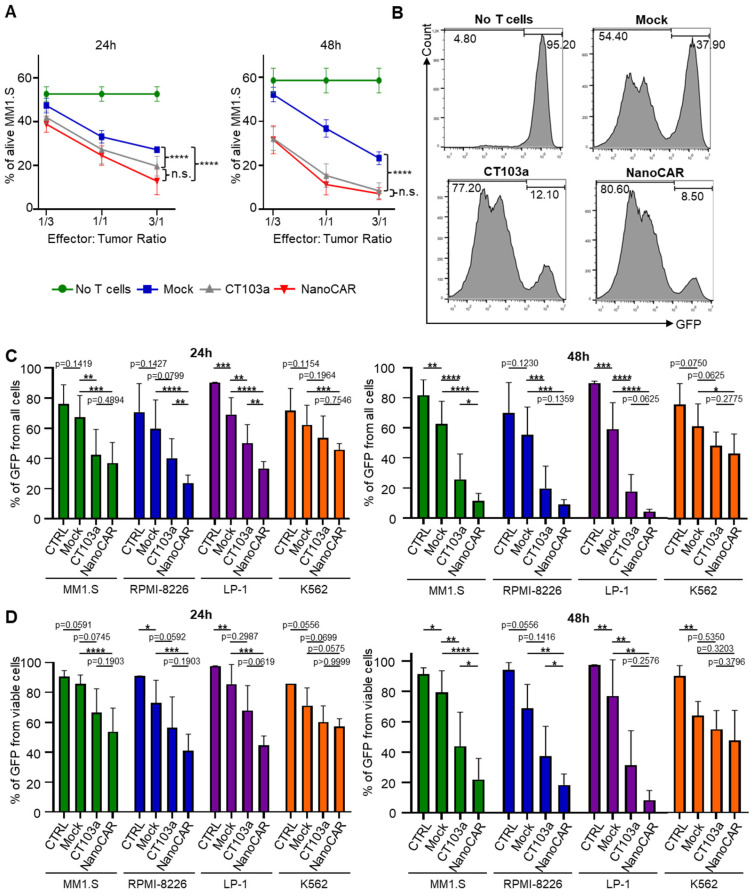
In vitro efficacy of CT103a and nanoCAR in killing BCMA+ MM cell lines. (**A**) In vitro cytotoxicity assays after 24 or 48 h of MM1.S-GFP at different effector-to-target (E:T) ratios (1:3, 1:1, 3:1). The mean value and SD from nine biological replicates are shown (n = 9). (**B**) Representative histograms from flow cytometry of in vitro cytotoxicity assays after 48 h of MM1.S-GFP at (E/T) ratio 1/1. (**C**,**D**) In vitro cytotoxicity assays (GFP from total cells C., GFP signal remaining from alive cells (**D**) after 24 h or 48 h of various GFP+-BCMA+ MM cell lines (MM1.S, RPMI-8226, and LP-1) or a GFP leukemia cell line (K562) with Mock T cells, CT103a, or nanoCAR in a 1:1 ratio. Results were presented as the mean value and SD of three replicates for three different donors (n = 3 for each of the 3 donors). n.s.: *p* > 0.05. *: *p* < 0.05. **: *p* < 0.01. ***: *p* < 0.001. ****: *p* < 0.0001, determined by Two-way ANOVA tests.

To evaluate the cytotoxic activity of the nanoCAR in comparison to CT103a and untransduced (Mock) activated T cells. We co-cultured each of them with the BCMA+ MM1.S-GFP cell line for 24 to 48 h, and tumor killing was assessed by quantifying the GFP signal using flow cytometry. We evaluated GFP expression at various effector-to-target (E:T) ratios of 1:3, 1:1, and 3:1. Both CAR-T cells significantly reduced the number of GFP+ tumor cells compared to Mock T cells after 24 to 48 h, with enhanced cytotoxicity observed at the later time point (Figure 2A,B, Supplementary Data Figure S2). To assess antigen specificity, CT103a, nanoCAR, and Mock T cells were co-cultured with various GFP+-BCMA+ MM cell lines (MM1.S, RPMI-8226, and LP-1) as well as with a GFP+-BCMA- leukemia cell line (K562) at a 1:1 ratio for 24 to 48 h. Robust cytotoxic activity was observed against BCMA+ targets for both CT103a and nanoCAR, particularly after 48 h (Figure 2C,D), even if K562 viability slightly decreased in co-culture. This could be explained by the basal IFNγ production of T/CAR-T cells (see Section 3.6). These findings support the antigen specificity of both CAR constructs, as they spare BCMA- cells while retaining cytotoxic activity against BCMA+ targets. Together, these results confirm the therapeutic potential of CT103a and nanoCAR in selectively targeting BCMA-expressing malignancies.

### 3.3. Persistence of CAR-T Killing Ability Following Repeated Antigen Challenges

**Figure 3:**
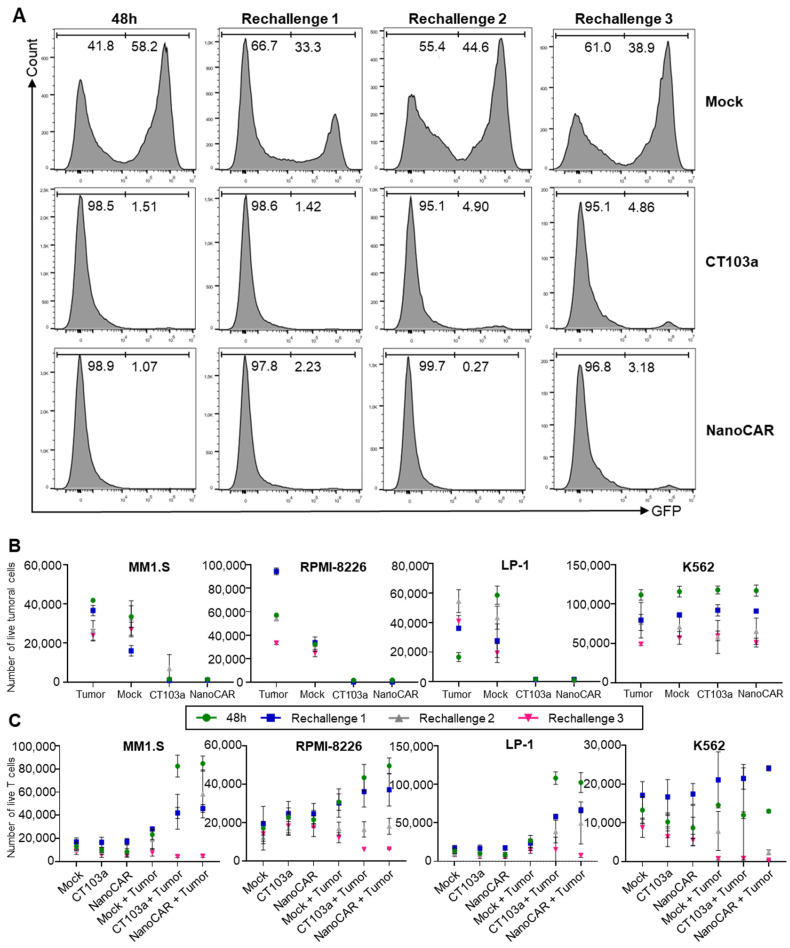
Persistence of CAR-T killing ability following repeated antigen rechallenges. (**A**) Representative histograms of GFP signal from flow cytometry of the killing capacity of CT103a and nanoCAR after four consecutive rechallenges every 2 days with MM1.S. (**B**) Evaluation by flow cytometry of killing capacity (GFP+ signal) of CT103a and nanoCAR by repeatedly rechallenging them four times, every 2 days, with various BCMA+ MM cell lines (MM1.S, RPMI-8226, and LP-1) or a BCMA- leukemia cell line (K562). Results were shown as the mean value and SD of two replicates for 2 different donors. (**C**) Evaluation by flow cytometry (CD3+ signal) of proliferation of CT103a and nanoCAR following four repeated challenges every 2 days with BCMA+ MM cell lines (MM1.S, RPMI-8226, and LP-1) or K562. Results were shown as the mean value and SD of two replicates for 2 different donors.

We demonstrated that both CT103a and nanoCAR were able to kill BCMA+ cells after 48 h. To further evaluate their functional persistence following repeated tumor challenges, we assessed the killing capacity of both CAR-T cells by repeatedly re-exposing them to their antigen and rechallenging them with various GFP+ cancer cell lines (MM1.S, RPMI-8226, LP-1, and K562). Specifically, Mock and CAR-T cells were incubated with tumor cell lines and rechallenged thrice, with intervals of 48 h. The killing capacity was evaluated by flow cytometry by quantifying the number of residual viable cancer cells, while CAR-T proliferation was measured by counting the number of live T cells. BCMA+ cell lines (MM1.S, RPMI-8226, and LP-1) were eradicated upon each rechallenge when co-cultured with either CT103a or nanoCAR, in contrast to the Mock group, which was incapable of killing all tumor cells or cancer cells incubated alone (Figure 3A,B). As a control, the BCMA- cell line K562 cells exhibited no significant cell death when co-cultured with T cells, CAR-T cells, or when cultured alone. In terms of proliferation, for each time point, both CT103a and nanoCAR demonstrated expansion when co-cultured with BCMA+ cells, compared to co-culture with K562 cells or in the absence of tumor cells. In contrast, Mock T cells exhibited minimal proliferation, both when cultured alone or in co-culture with any tumor cell type (Figure 3C). While these results showed that both CT103a and nanoCAR retained functional activity across several short-term rechallenges, we acknowledged that our assay, consisting of three consecutive 48 h stimulations, does not capture long-term CAR-T-cell exhaustion. Therefore, we refrain from making claims regarding differences in exhaustion between the two constructs. We concluded that, within the limitations of this assay, both CT103a and nanoCAR maintained functional persistence over repeated exposure, indicating their potential for sustained tumor control.

### 3.4. CAR-T-Cell Differentiation into Central Memory Subsets with Enhanced CD107a Surface Expression

**Figure 4:**
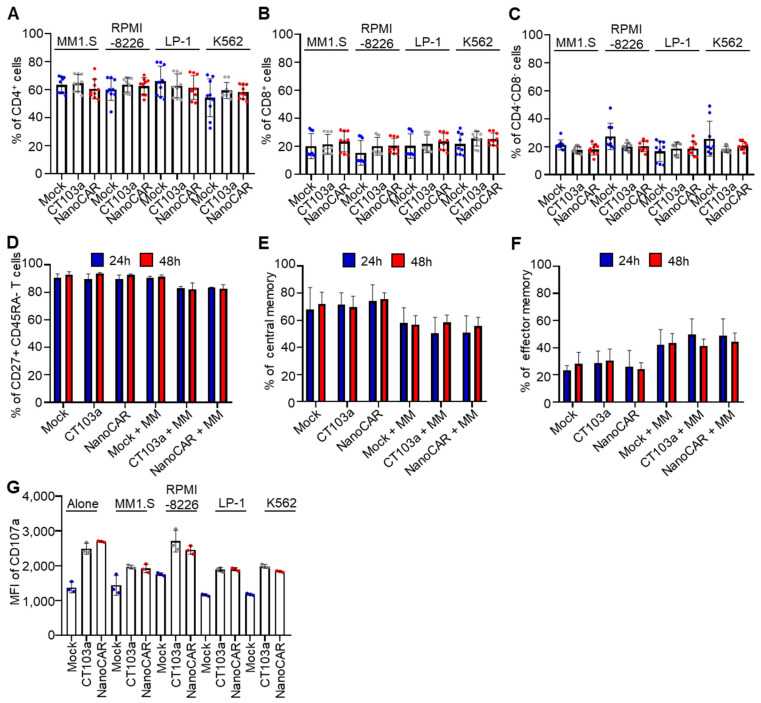
CAR-T-cell differentiation into central or effector memory subsets with enhanced CD107a surface expression in vitro (**A**–**C**). Evaluation of CD4+ (**A**), CD8+ (**B**), and CD4-CD8- (**C**) levels after 24 h of co-culture with MM1.S, RPMI-8226, LP-1, and K562. The mean value and SD from 3 biological replicates of 3 different donors were shown (n = 3 for each donor). (**D**) Evaluation by flow cytometry of memory phenotype (CD27+ CD45RA-) after 24 h to 48 h of co-culture with MM1.S followed by the analysis of central CD62L+ (**E**) or effector CD62L- (**F**) rates after 24 h to 48 h of co-culture of non-activated T cells, Mock T cells, CT103a, and nanoCAR. (**G**) Expression of the degranulation marker CD107a. Evaluation of CD107a MFI signal after 6 h of co-culture with MM1.S, RPMI-8226, LP-1, and K562. The mean value and SD from 3 independent experiments, utilizing a total of 3 different donors, are shown. No significance using Mann–Whitney test.

Before analyzing differentiation subsets, we evaluated the levels of CD4+, CD8+, and CD4-CD8- after 24 h of co-culture with MM1.S, RPMI-8226, LP-1, and K562. Overall, CD4+ levels (Figure 4A) remained consistent across the groups, as were CD8+ (Figure 4B) and CD4-CD8- levels (Figure 4C). Additionally, the literature indicates that CD4+ levels represented approximately 60%, CD8+ levels ranged between 20 and 30% as CD4-CD8- levels, aligning with the typical distribution observed in the T-cell populations herein [28].

To assess differentiation of CT103a and NanoCAR co-cultured with MM1.S for 24 to 48 h, we evaluated the expression of CD27, CD45RA, and CD62L using flow cytometry (Supplementary Data Figure S3A). Mock T cells, CT103a, and nanoCAR presented a high frequency of CD27+-CD45RA- cells (Figure 4D), indicating a memory T-cell phenotype. Within this memory population, we assessed the CD62L expression to discriminate central memory (TCM, CD62L+) and effector memory (TEM, CD62L-) subsets (Figure 4E,F). TCM cells are associated with long-term persistence and lymph node homing, whereas TEM cells provide rapid effector functions in peripheral tissues. At 24 h, CAR-T cells co-cultured with tumor cells displayed a higher proportion of TEM cells compared to Mock or CAR-T cells cultured without targets, consistent with antigen-driven activation and cytotoxic engagement. By 48 h, the TEM population showed a slight decrease, suggesting that, after initial antigen clearance, CAR-T cells can partially re-establish a memory-like phenotype. In contrast, Mock and CAR-T cells maintained in culture without tumor cells preserved a predominantly TCM profile at both 24 and 48 h, underscoring the importance of antigen encounter in promoting effector differentiation. The expression of the degranulation marker CD107a was assessed 6 h after co-culture with MM1.S, RPMI-8226, LP-1, and K562 (Figure 4G, Supplementary Data Figure S3B) by flow cytometry. Data showed higher, but non-significant, expression in CT103a and nanoCAR compared to Mock T cells, even for K562 cells. This could be explained by the basal CD107a expression observed in activated CAR-T cells, which may occur independently of the antigen recognition.

### 3.5. CT103a and nanoCAR Produce Cytokines When Co-Cultured with MM Cell Lines

**Figure 5:**
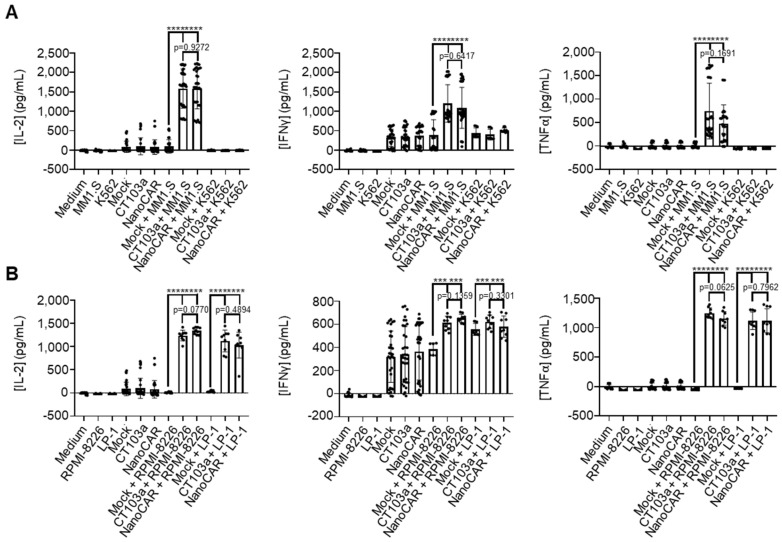
CT103a and nanoCAR produce cytokines when co-cultured with MM cell lines. Measurements of cytokine production (IL-2, IFNγ, TNFα) after 24 h of co-culture with MM1.S and K562 cells (**A**), or with RPMI-8226 or LP-1 cells (**B**). Results expressed as mean value and SD from five independent experiments, utilizing a total of seven different donors in triplicate (n = 3 for each of the seven donors). ***: *p* < 0.001. ****: *p* < 0.0001, determined by Mann–Whitney test.

The production of cytokines is a key aspect of CAR-T-cell characterization. We measured the levels of IL-2, IFNγ, and TNFα after 24 h of co-culture with either MM1.S or K562 cells (Figure 5A), as well as RPMI-8226 or LP-1 (Figure 5B). When Mock or CAR-T cells were co-cultured with the BCMA- K562 cell line, only minimal amounts of IFNγ were detected, and neither IL-2 nor TNFα was produced. In contrast, those co-cultured with MM1.S resulted in a significant increase in the cytokine production of CT103a and nanoCAR, although the difference between the two CAR constructs was not statistically significant. This specific cytokine production of CT103a and nanoCAR underscores their increased functional activity in response to BCMA+ targets.

### 3.6. Gene Expression Studies of CT103a and nanoCAR Co-Cultured with MM Cells

To gain deeper insights into CAR-T activity, we performed bulk RNA sequencing and compared the transcriptional profiles across the different conditions (CT103a, nanoCAR, Mock) after incubation with MM1.S (Supplementary Data Figure S4). Given that critical biological events occur rapidly after the encounter between CAR-T cells and target cells, we selected early time points (0, 4, and 16 h post-incubation) for this analysis. We used Mock T cells as a negative control and CT103a as a positive control.

**Figure 6:**
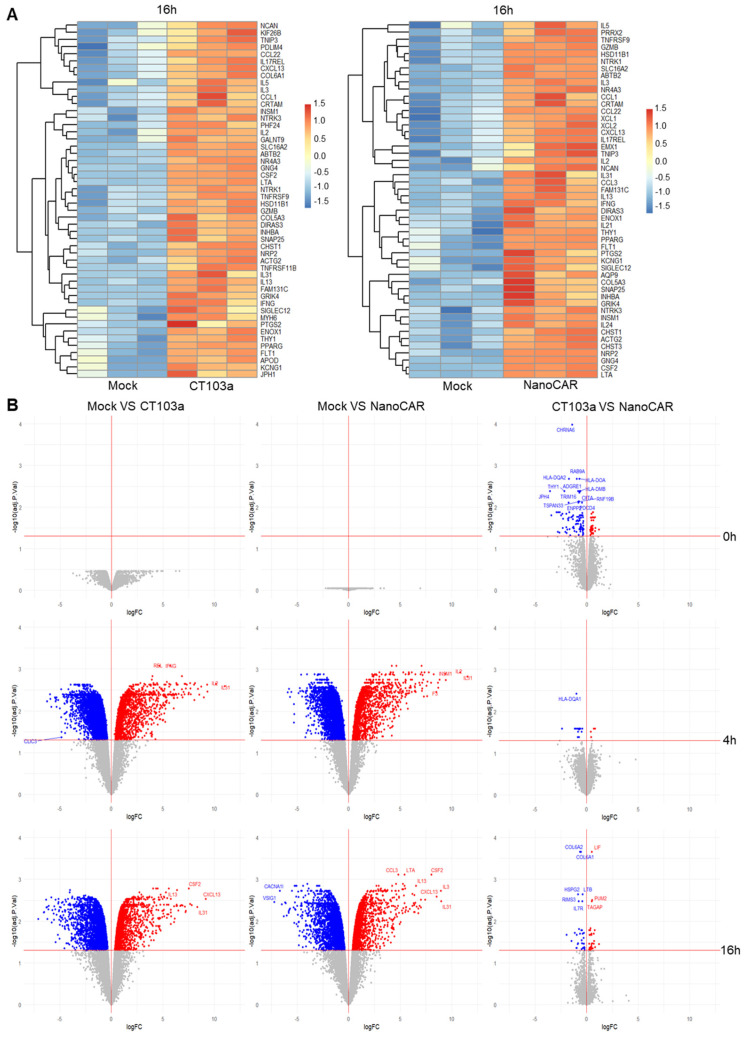
CT103a and nanoCAR express similar set of genes and pathways (bulk RNA seq) when co-cultured with MM cells (n = 3 independent donors). (**A**) List the top 50 overexpressed genes, based on adjusted *p*-value, of CT103a and nanoCAR compared to activated Mock T cells for three different donors using heatmaps. (**B**) List of the significant upregulated and downregulated genes of 0 h, 4 h, and 16 h post-co-culture with MM1.S using Volcano plots. These plots compared CT103a with Mock used as a negative control, nanoCAR with Mock used as a negative control, and nanoCAR with CT103a used as a reference.

To explore the transcriptional responses of CT103a and nanoCAR, we first visualized the top 50 overexpressed genes of both CAR-Ts compared to Mock at 16 h using heatmaps (Figure 6A). Both CAR-T constructs demonstrated upregulation of key genes associated with T-cell activation and proliferation (e.g., *IL17REL*, *TNFRS9* (*4-1BB*)) or effector functions (e.g., *IL5*, *IL2*, *IFNG*). Complementing this analysis, volcano plots revealed minimal differences between CT103a and nanoCAR across all time points (0 h, 4 h, 16 h). In contrast, both CAR-T-cell types exhibited a significant increase in the number of upregulated and downregulated genes at 4 h and 16 h compared to Mock T cells, consistent with CAR-specific activation (Figure 6B).

We examined genes (Supplementary Data Figure S5) significantly up- or downregulated by nanoCAR compared with CT103a at 0 h, 4 h, and 16 h to capture their transcriptional profiles and potential differences. At 0 h, NanoCAR upregulated *IFITM2*, a viral restriction factor, whereas CT103a upregulated *NECTIN1* and *IFIH1*, potentially facilitating viral entry and sensing, which may explain differences in transduction efficiency without necessarily reflecting functional cytotoxicity. CT103a also showed early upregulation of MHC class II and antigen presentation genes, including multiple HLA isoforms and *CD74*, suggesting enhanced transcriptional readiness for antigen presentation, although in the context of short-term co-culture in 96-well plates, this likely reflects transcriptional activity rather than functional antigen presentation.

Cytokine signaling and differentiation pathways also differed. CT103a upregulated *IL17F*, *CXCL10*, and *LIF*, as well as Wnt and differentiation-related genes (e.g., *WNT5B*, *TCF7*, *RXRA*, *FOXP1*), consistent with memory-like and differentiation-prone transcriptional programs. NanoCAR, in contrast, upregulated *OSM* and components of the IL-18 pathway. Both CARs modulated cytoskeleton and adhesion genes, but CT103a exhibited a stronger signature for migration and extracellular matrix interactions, potentially reflecting enhanced early physical engagement with tumor cells.

Metabolic and transcriptional regulation programs were distinct. CT103a upregulated mitochondrial and metabolic genes (*UCP2*, *DHCR24*, and *ABCA2*), while nanoCAR upregulated *GSTK1*, *NUCB2*, and *ACSL1*. Transcription factors and epigenetic regulators differed as well, with nanoCAR upregulating *FOXP3*, *SCML4*, and *RFX5*, and CT103a upregulating *FOXP1*, *HDAC7*, *CHD8*, and *ZMIZ2*, reflecting distinct regulatory networks.

Collectively, these data demonstrated that nanoCAR and CT103a activated different transcriptional programs during direct tumor cell interactions. CT103a showed stronger antigen presentation, cytokine signaling (excluding IL-18), and Wnt and metabolic gene signatures, whereas NanoCAR exhibited protective or regulatory gene expression. These differences highlighted that transcriptional differences do not necessarily predict overall functional superiority.

Gene set enrichment analysis (GSEA) provided further insights into CAR-T-cell activation. Hallmark (Supplementary Data Figure S6A) and KEGG (Supplementary Data Figure S6B) pathway analyses revealed significant enrichment of pathways related to cytokine production, cytokine–cytokine interactions, and proliferation in CT103a and nanoCAR compared to Mock at 4 h. This enrichment reflected CAR-specific functionality. However, these pathways were no longer differentially expressed by 16 h, likely due to basal cytokine production by pre-activated Mock T cells masking CAR-specific effects. These findings suggested that cytokine-related signaling at early time points predominantly arises from CAR-specific mechanisms, while the distinction diminished over time due to baseline activation in Mock cells. Overall, while CT103a and nanoCAR demonstrated similar transcriptional profiles, their distinct gene signatures relative to Mock revealed shared mechanisms of antigen-specific activation.

### 3.7. CT103a and nanoCAR Can Eliminate Tumor Cell In Vivo

**Figure 7:**
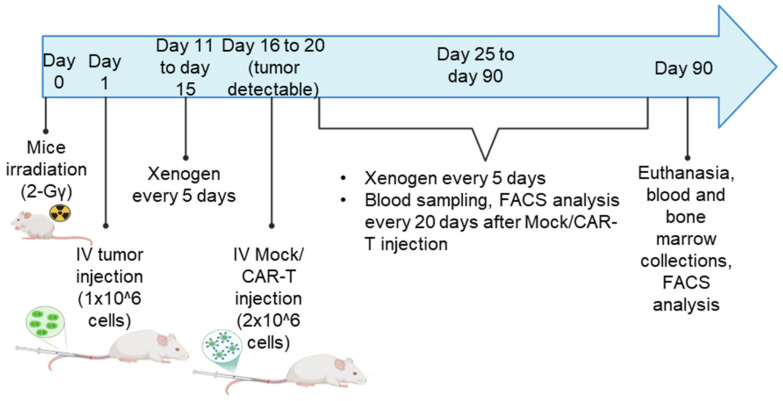
Workflow diagram of the in vivo experiment using an NSG mouse model, showing the timing of Mock T cell or CAR-T-cell injections, tumor monitoring by bioluminescence, blood sampling, and final euthanasia for sample collection.

**Figure 8:**
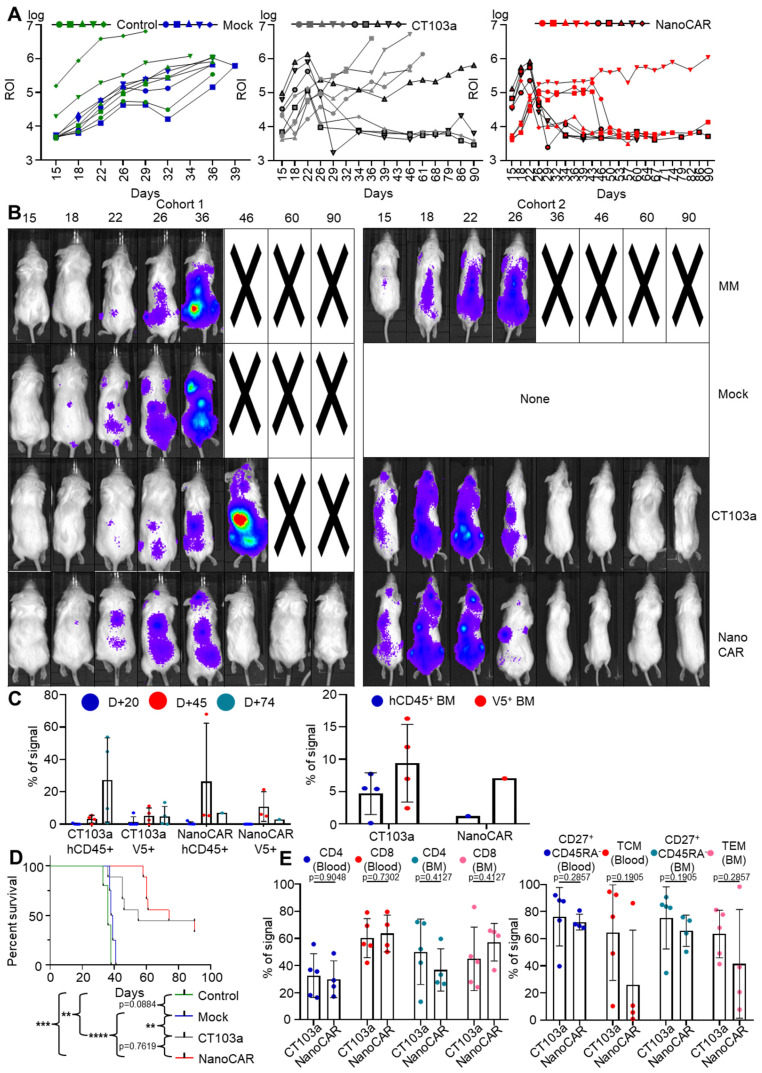
CT103a and nanoCAR-T cells display anti-MM activity in vivo in a murine model. (**A**) Representative regions of interest (ROI) from bioluminescence imaging illustrating tumor progression over time. (**B**) Representative bioluminescent images of mice from each treatment group (MM, Mock, CT103a, and nanoCAR) at different timepoints. (**C**) Flow cytometry analysis of immune reconstitution in peripheral blood and BM of mice from the second cohort by analyzing frequency of human CD45+ (hCD45) cells and V5+ (CAR-T+) cells among hCD45+populations. (**D**) Kaplan–Meier survival curves of treated mice showing MM-related mortality. Results are presented as the mean value and standard deviation (SD) from several independent experiments. **: *p* < 0.01. ***: *p* < 0.001. ****: *p* < 0.0001, determined by Mantel-Cox log-rank test. (**E**) Flow cytometry analysis of immune reconstitution in peripheral blood and BM of CD4-CD8 subsets, and TCM or ECM phenotypes. Left: distribution of CD4+ and CD8+ T cells. Right: frequency of CD62L+ cells among CD27+CD45- memory T cells. Each dot represents one mouse; bars indicate median values.

We examined the anti-tumor efficacy of both CT103a and nanoCAR-T cells in a NSG mouse model using the Xenogen IVIS 200 imaging system and flow cytometry (Supplementary Data Figure S7). Mice were intravenously injected with 1 × 10^6^ MM1.S-GFP-Luc and subsequently treated with 2 × 10^6^ Mock T cells, CT103a, or nanoCAR T cells via intravenous injection 16 to 19 days after tumor inoculation once tumor signals became detectable by bioluminescence. Tumor proliferation was monitored by bioluminescence; blood samples were collected every 20–30 days following Mock or CAR-T-cell injection. Mice were euthanized on day 90 to collect blood and bone marrow for further analysis (Figure 7). In cohort 1, all the mice injected with MM1.S-GFP-Luc cells and left untreated or treated with Mock T cells succumbed to disease within 30–40 days, confirming the aggressiveness of the model (Figure 8A,B, Supplementary Data Figure S8A). CT103a-treated mice died by day 36 to 46. In contrast, all four mice treated with nanoCAR-T cells survived beyond day 90, with three out of four mice being completely tumor-free, and one mouse showing residual disease. In cohort 2, both CT103a and nanoCAR-T treatments induced complete tumor regression within 10 days of CAR-T-cell infusion (Figure 8A,B, Supplementary Data Figure S8B). Mice were subsequently monitored for long-term survival. Four out of five nanoCAR-T-treated mice had signs of graft-versus-host disease and died before day 90. Among CT103a-treated mice, two also developed signs of graft-versus-host disease with signs of ascites and jaundice. A two-way ANOVA test was performed on the combined bioluminescence data from both cohorts up to day 34. This analysis revealed a significant effect of treatment condition (*), indicating that treatment differences became detectable beginning at this stage. At day 20 after CAR-T infusion, peripheral blood analysis showed very low or undetectable levels of hCD45^+^ cells and consequently no measurable CAR-T cells. By day 45 after CAR-T infusion, mice displayed low amounts of hCD45^+^ cells, with low levels of V5^+^ CAR-T cells (0.5–15%). By day 74 after CAR-T infusion (corresponding to day 90, the day of euthanasia), the surviving mice exhibited variable amounts of hCD45^+^ cells, while V5^+^ CAR-T cells remained low (0.5–10%) (Figure 8C). In addition, at day 90, the surviving mice presented both hCD45^+^ and V5^+^ CAR-T cells in their BM (Figure 8C). For mice who died before day 90, the amount of hCD45^+^ cells was generally high (25–70%) in the peripheral blood with low amounts of V5^+^ CAR-T cells, and similar hCD45^+^ and V5^+^ cells in the BM (Supplementary Data Figure S9B). For the Kaplan–Meier survival curves, the global mortality is shown for all mice with no significance between the CAR-treated mice (Figure 8D). However, the MM-related mortality showed significantly superior myeloma responses in the nanoCAR-T-treated mice (Supplementary Data Figure S9A). We next evaluated CD4 and CD8 T-cell subsets, as well as the proportion of CD62L+ cells among CD27+CD45RA- memory population (Figure 8E). We found no significant differences in phenotype between CT103a and nanoCAR-T cells. These findings suggest that, while both CT103a and nanoCAR-T cells display potent anti-MM activity, but they may additionally induce xenoreactivity against murine tissues in our in vivo studies.

## 4. Discussion

Our study provided a detailed characterization of two anti-BCMA CAR-T therapies, CT103a (ScFv-based) and nanoCAR (VHH-based), for MM treatment. Despite their structural difference, both CAR constructs demonstrated comparable cytotoxic activity, persistence, and transcriptional behavior. Previous studies have highlighted intrinsic advantages of VHH domains, including their small size, stability, and lack of VL-VH pairing, which reduces misfolding, aggregation, and tonic signaling. Their high solubility, epitope accessibility, and lower immunogenicity may also support robust CAR expression and persistence. These structural features are particularly advantageous in multispecific CAR designs; several studies have shown that nanoCAR-T cells demonstrated comparable tumor killing efficacy, and their compact configuration enables more efficient antigen engagement and enhanced therapeutic performance in bispecific settings [29,30,31,32,33,34]. Together, these findings support VHH-based CARs as promising and flexible alternatives to conventional scFv-CAR-T designs.

Both CT103a and nanoCAR specifically targeted BCMA+ MM cell lines (MM1.S, RPMI-8226, and LP-1), while sparing BCMA^−^ K562 cells. This activity was maintained over prolonged co-culture and repeated antigen exposures. Moreover, key cytokines, including IL-2, TFNα, and IFNγ, were produced in response to BCMA^+^ targets, confirming antigen-dependent functionality of both CAR-T constructs [35,36].

T-cell phenotyping showed that all CAR-T and Mock T cells displayed a memory-like profile (CD27^+^CD45RA^−^) compared to non-activated PBMC-derived T cells, with CAR transitioning dynamically between TCM and TEM. An early rise in TEM at 24 h followed by a return toward TCM at 48 h suggested active engagement followed by recovery, consistent with functional resilience. Moreover, their functional readiness was further reinforced by the expression of degranulation markers such as CD107a. These characteristics were essential for effective CAR-T treatments, providing insights into how CAR-T cells maintain robust and extended antitumor activity over time [37].

Transcriptional analyses revealed largely shared early activation profiles, with both CAR-T upregulating genes involved in T-cell activation, proliferation, and effector functions. GSEA confirmed enrichment of common pathways, such as cytokine production and cytokine–cytokine interactions at 4 h, which largely diminished by 16 h, possibly reflecting baseline cytokine production in pre-activated T cells. Despite these similarities, each CAR also displayed distinctive transcriptional tendencies. CT103a upregulated MHC class II-associated genes, Wnt-related and metabolic genes, and an early immunomodulatory and metabolic activation program. In contrast, nanoCAR selectively induced IL-18-related pathways, suggesting a more protective or modulatory transcriptional response. Together, these data highlighted that while both constructs engage widely similar activation pathways upon tumor direct contact, they diverged in specific regulatory and metabolic programs, without implying functional superiority of either CAR construct.

In vivo, both CAR-T therapies demonstrated potent anti-MM activity. The long-term survival and minimal residual disease observed in nanoCAR-T-treated mice highlight the therapeutic potential of VHH-based CAR-T designs, particularly in aggressive MM models. Notably, nanoCAR-T cells displayed prolonged survival in both the BM and peripheral blood. In contrast, the limited peripheral circulation in CT103a-treated mice suggests distinct capacities in persistence and in vivo expansion between the two CAR constructs. While cohort 2 further supports the potent anti-tumor activity of both CAR-T therapies, the occurrence of ascites and signs of graft-versus-host disease in several CAR-T-treated mice may suggest possible xenoreactivity against murine tissues. Previous studies showed that engrafted T-cells in NSG mice may induce xenogeneic graft-versus-host disease that is dependent on the expression of murine MHC class I and II molecules [38,39]. Together with phenotypic and functional profiling, these results underscore the potential of VHH-based CAR-T therapies as a promising strategy for the treatment of BCMA+ MM.

In conclusion, our results showed that CT103a and nanoCAR are promising CAR-T treatments for BCMA^+^ MM. Both constructs demonstrated significant cytotoxic activity, persistence, and favorable transcriptional profiles, indicating their potential as effective therapeutic approaches. Notably, compared to traditional scFvs, the integration of nanobodies, such as the VHH Nb17, in CAR designs could provide several advantages, including smaller size, higher stability, and the ability to target distinct epitopes or antigens with reduced steric hindrance. Similar findings were reported in studies using VHH-based nanoCAR-T cells targeting CD19, which showed comparable cytotoxicity potency and persistence to conventional scFv-based CAR-T cells, further supporting the feasibility of nanobody integration in CAR design [29]. These benefits could improve CAR-T cell targeting accuracy, increasing their overall efficacy, especially in full tumor microenvironments. Future research could focus on optimizing bispecific CAR-T cells, which would target multiple antigens to reduce the possibility of antigen escape and increase treatment durability [40,41]. Moreover, exploring the integration of nanobodies in combination with immune checkpoint inhibitors or other immune-modulating treatments could enhance CAR-T functionality, overcoming limitations like immune resistance. Finally, the potential for engineering CAR-T cells with a more defined “memory-like” phenotype could offer strategies to improve long-term persistence, reducing the need for repeated treatments [42]. These next steps would move beyond initial proof-of-concept studies to more clinical and mechanistic understanding, facilitating the translation of these promising CAR-T therapies into the clinic.

## Data Availability

The data presented in this study are available from the corresponding author upon reasonable request.

## Supplementary Materials

The following supporting information can be downloaded at: https://www.mdpi.com/article/10.3390/cells14241944/s1.

## Abbreviations

The following abbreviations are used in this manuscript:

MM	Multiple myeloma
BCMA	B-cell maturation antigen
BM	Bone marrow
CAR	Chimeric antigen receptor
Cilta-cel	Ciltacabtagene autoleucel
FDA	Food and Drug Administration
GSEA	Gene set enrichment analysis
IL-2	Interleukin-2
MOI	Multiplicity of infection
ScFv	Single-chain variable fragment
SdAb	Single domain antibody
SD	Standard deviation
TCM	Central memory T cell
TEM	Effector memory T cell
VHH	Heavy chain variable domains
