# Supplementary material for: Comparing a Novel Anti-BCMA NanoCAR with a Conventional ScFv-Based CAR for the Treatment of Multiple Myeloma
Source: Cells. 2025 Dec 8;14(24):1944. doi: 10.3390/cells14241944 (PMC12731311; doi:10.3390/cells14241944)
Supplement: Supplementary file 1 [file cells-14-01944-s001.zip › cells-3973035-supplementary.pdf]

cells

MDPI

Supplementary Data

Table S1 Sequences of CT103a and Nb17

**DNA sequence of CT103a**
gccttaccagtgaccgccttgctcctgccgctggcctt-<br>gctgctccacgccgccaggccgggcaaacccatccctaaccctctgctgggcctggacagcaccgacatccagatgacccag<br>agcccttctagcctgtctgctagcgtgggcgatagagtgacaattacctgcagagcttctca-<br>gagcatcagctcttacctgaactggtaccaacaaaagcctggcaaggcccctaagctgctgatctacgccgcctctagcctcc<br>agagtggagttccaagccgcttcagcggctctggcagcggaactgacttcaccct-<br>gaccatcagctccctgcagcctgaagatttcgctacctactactgccagcagaagtacgacctgctgacattcggcggcggca<br>caaaggtggaaatcaagggcagcac-<br>cagcggaagcggcaaacctggcagcggcgagggcagcacaaagggccaactgcagctgcaggagagcggtcctggcct<br>ggtcaagcctagcgagacactgtccttgacatgcacggtgtccggag-<br>gaagcatctcaagcagctcttattactggggatggatccggcagcctccaggaaagggcctcgagtggatcggctccatcag<br>ctacagcggcagcacctactacaacccaagcctgaagagccgggtgaccatctctgtcga-<br>caccagcaagaatcagtttagcctgaaactgagcagcgtgacagccgctgataccgctgtgtactactgcgccagagacaga<br>ggcgacaccatcctggacgtgtggggccagggcaccatgg-<br>tgacagtgtcctctgcgaagcccaccacgacgccagcgccgcgaccaccaacaccggcgcccaccatcgcgtcgcagcccc<br>tgtccctgcgcccagaggcgtgccggccagcggcgggggggcgcagtgca-<br>cacgagggggctggacttcgcctgtgatatctacatctgggcgcccttggccgggacttgtggggtccttctcctgtcactggt<br>tatcaccctttactgcaaacggggcagaaagaaactcctgtatatattcaaacaaccat-<br>ttatgagaccagtacaaactactcaagaggaagatggctgtagctgccgatttccagaagaagaagaaggaggatgtgaac<br>tgagagtgaagttcagcaggagcgcagacgcccccgcgtaccagcagggccagaaccagctc-<br>tataacgagctcaatctaggacgaagagaggagtacgatgttttggacaagagacgtggccgggaccctgagatgggggg<br>gaaagccgagaaggaagaaccctcaggaaggcctgtacaatgaactgca-<br>gaaagataagatggcggaggcctacagtgagattgggatgaaaggcgagcgccggaggggcaaggggcacgatggcc<br>tttaccagggtctcagtacagccaccaaggacacctacgacgcccttcacatgcaggccctgccccctcgc
**DNA and amino acid sequences of Nb17**
cag gtg cag ctg cag gag agc ggc ggc ggt ctg gtt caa gcg ggc ggt agc ctg cgt ctg<br>Q V Q L Q E S G G G L V Q A G G S L R L<br><br>agc tgc gcg gcg agc ggt cgt acc ttc acg atg ggt tgg ttc cgt cag gcg ccg ggt aaa<br>S C A A S G R T F T M G W F R Q A P G K<br>gag cgt gaa ttc gtt gcg gcg atc agc ctg agc ccg acc ctg gcg tac tat gcg gag agc<br>E R E F V A A I S L S P T L A Y Y A E S<br>gtt aag ggc cgt ttt acc att agc cgt gac aac gcg aaa aac acc gtg gtt ctg caa atg<br>V K G R F T I S R D N A K N T V V L Q M<br>aac agc ctg aag ccg gaa gat acc gcg ctg tac tat tgc gcg gcg gat cgt aag agc gtt<br>N S L K P E D T A L Y Y C A A D R K S V<br>atg agc att cgc ccg gat tat tgg ggt caa ggc acc cag gtc acc gtc tcc tca<br>M S I R P D Y W G Q G T Q V T V S S

 https://doi.org/10.3390/cells14241944

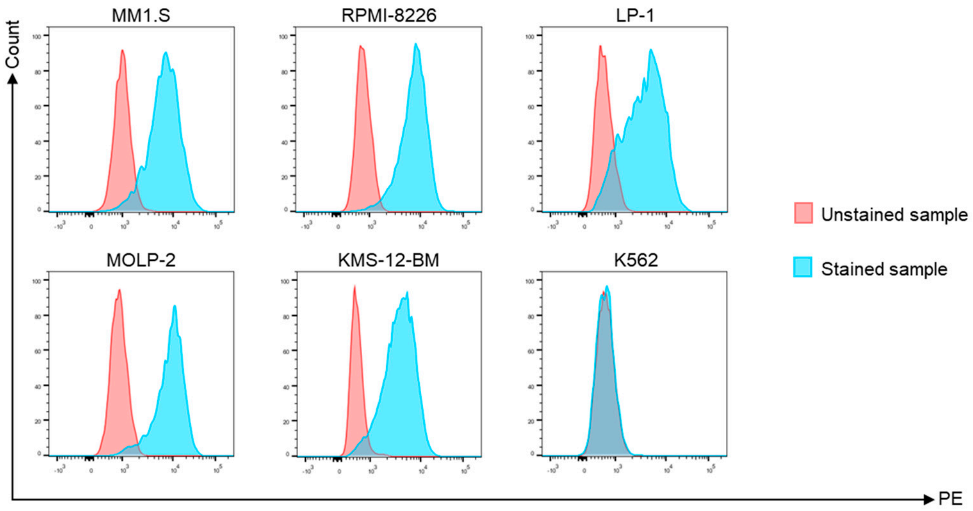

Figure S1 Quantification by flow cytometry of the number of cell surface BCMA molecules on K562 and MM cell lines: MM1.S, RPMI-8226, LP-1, MOLP-2 and KMS-12-BM (n = 14 independent experiments).

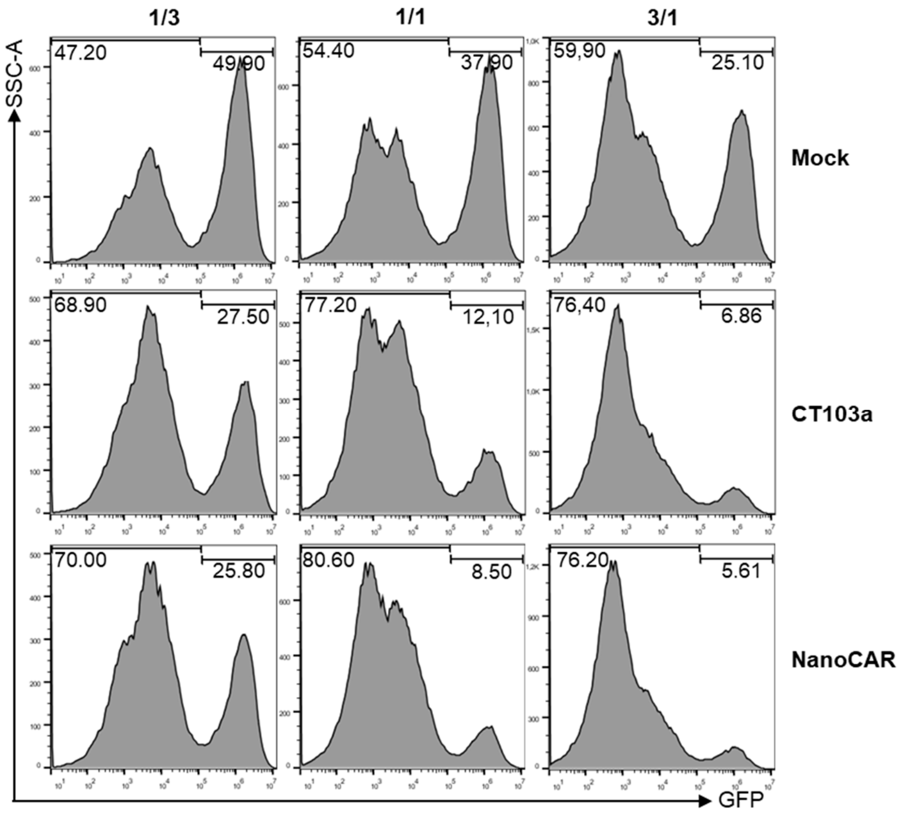

Figure S2 In vitro efficacy of CT103a and nanoCAR in killing BCMA+ MM cell lines. Representative histograms from flow cytometry of in vitro cytotoxicity assays after 48 hours of MM1.S-GFP at different effector-to-target (E/T) ratios (1/3, 1/1, 3/1).

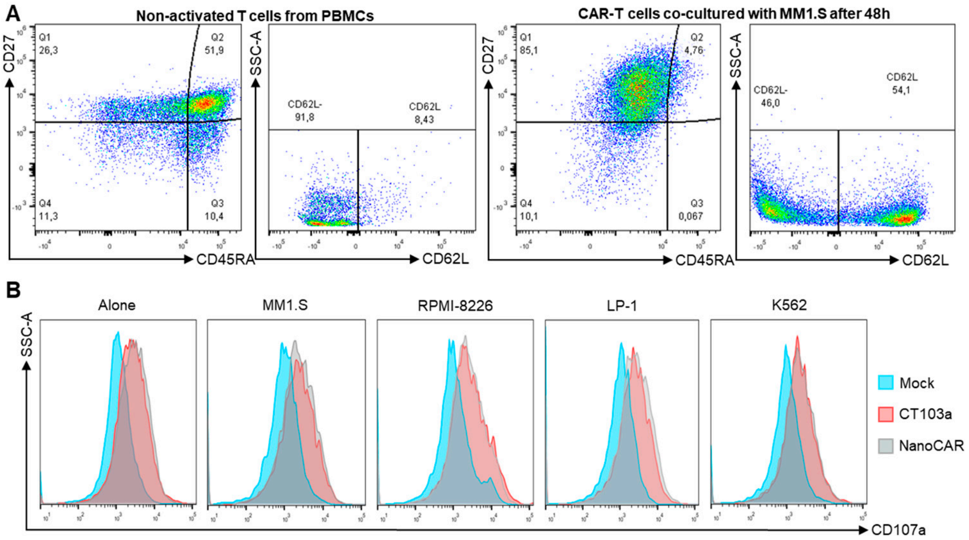

Figure S3 CAR-T cell differentiation into central or effector memory subsets with enhanced CD107a surface expression in vitro A. Flow cytometry gating strategy to evaluate the frequency of central and effector memory CAR-T cells during co-culture with MM1.S compared to non-activated PBMC-derived T cells. B. Evaluation by flow cytometry of the degranulation marker CD107a after 6 hours of co-culture with MM1S, RPMI-8226, LP-1 and K562.

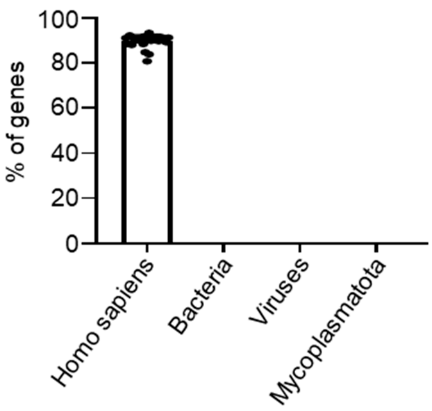

Figure S4 Gene expression studies of CT103a and nanoCAR co-cultured with MM cells. Representative histogram showing the bulk RNA seq analysis were perfomed without any gene contamination from bacteria, viruses, or mycoplamatota.

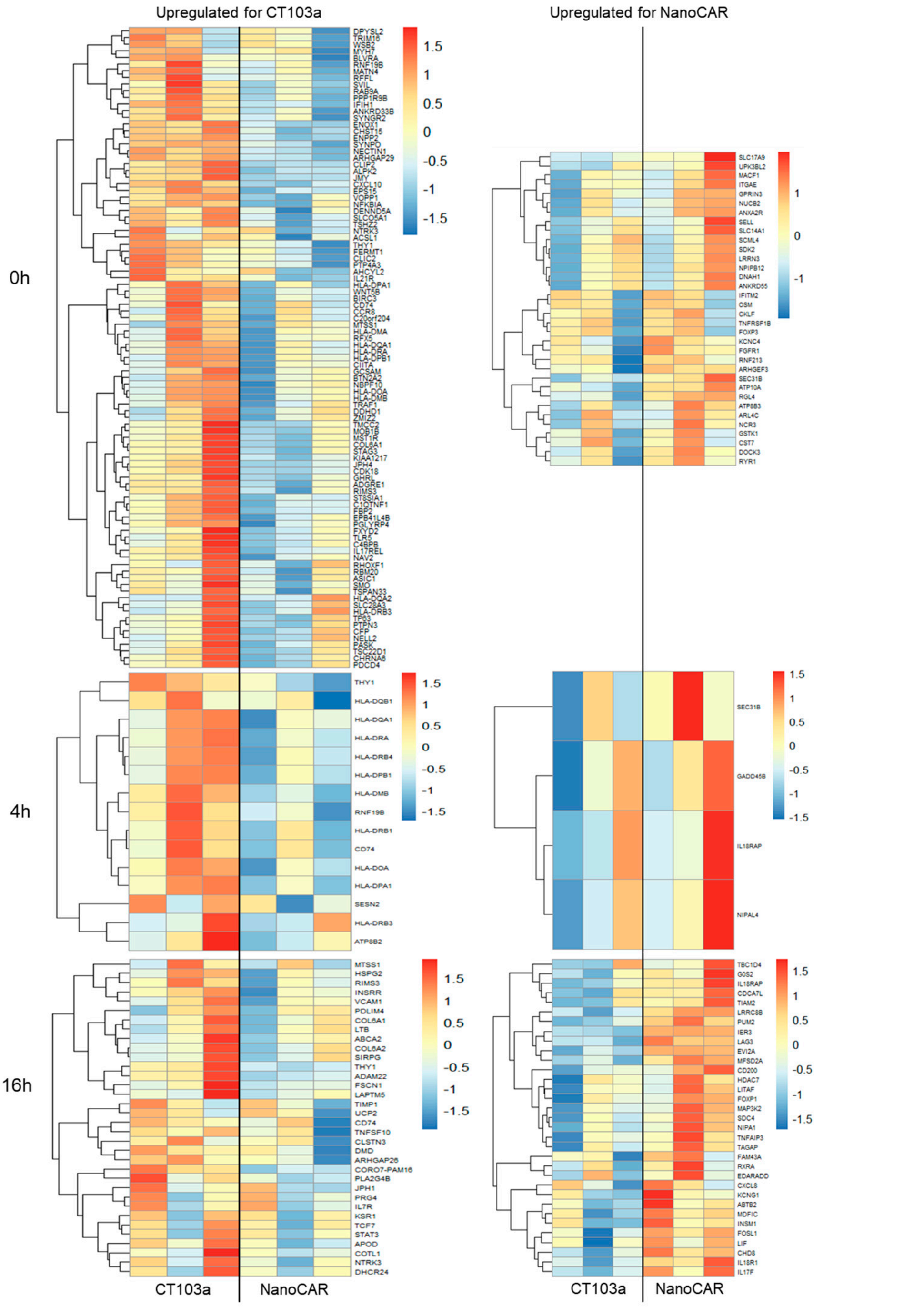

Figure S5. List the upregulated genes from CT103a (left column) or NanoCAR (right column) at 0h, 4h, and 16h. At 0h, there are 96 upregulated genes for CT103a and 34 for NanoCAR. At 4h, there are 15 upregulated genes for CT103a and 4 for NanoCAR. At 16h, there are 34 upregulated genes for CT103a and 33 for NanoCAR.

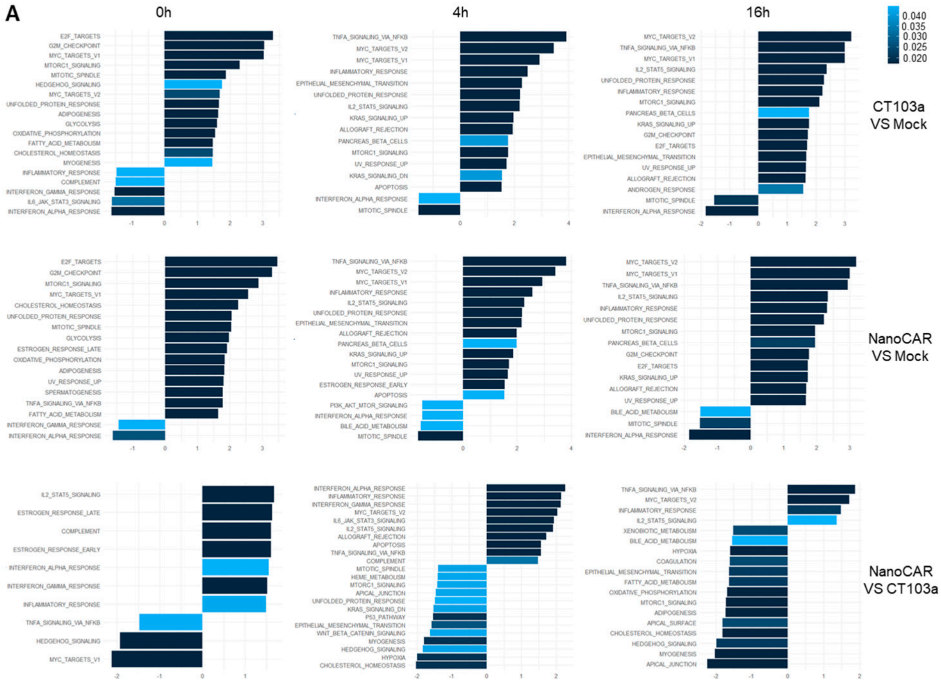

Figure S6 A. NanoCAR and CT103a express similar set of genes and pathways (bulk RNA seq) when co-cultured with MM cells. Gene set enrichment analysis in Hallmark to illustrate enriched upregulated and downregulated pathways after of CT103a or nanoCAR compared to the negative control Mock after 0h, 4h and 16h of co-culture with MM1.S cells. Results were shown using three donors (n = 3), grey barrs representing n.s. pathways, significant pathways were represented using a gradient of blue.

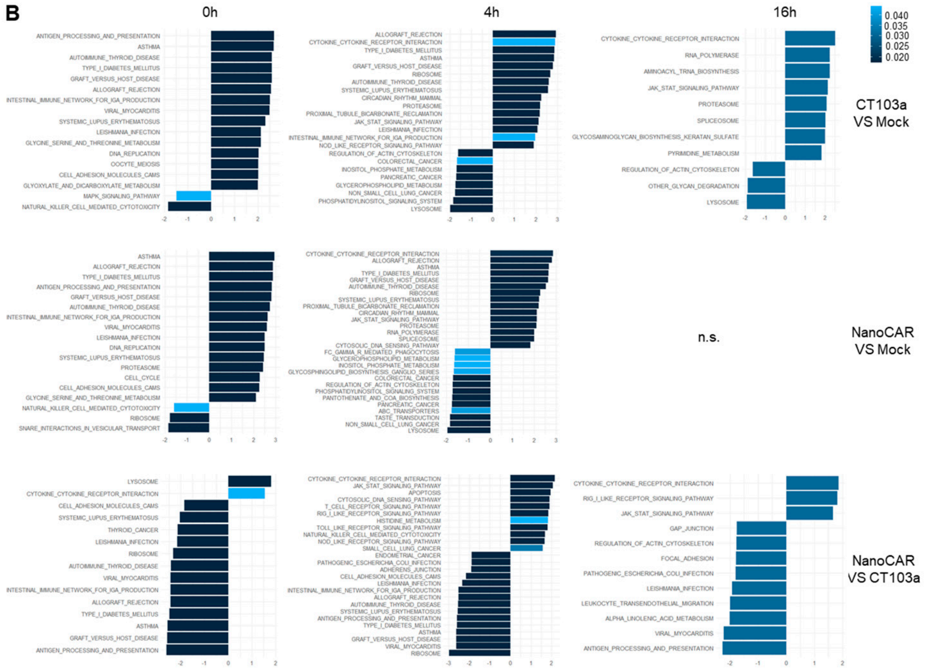

Figure S6 B. NanoCAR and CT103a express similar set of genes and pathways (bulk RNA seq) when co-cultured with MM cells. Gene set enrichment analysis in KEGG to illustrate enriched upregulated and downregulated pathways after of CT103a or nanoCAR compared to the negative control Mock after 0h, 4h and 16h of co-culture with MM1.S cells. Results were shown using three donors (n = 3), grey barrs representing n.s. pathways, significant pathways were represented using a gradient of blue.

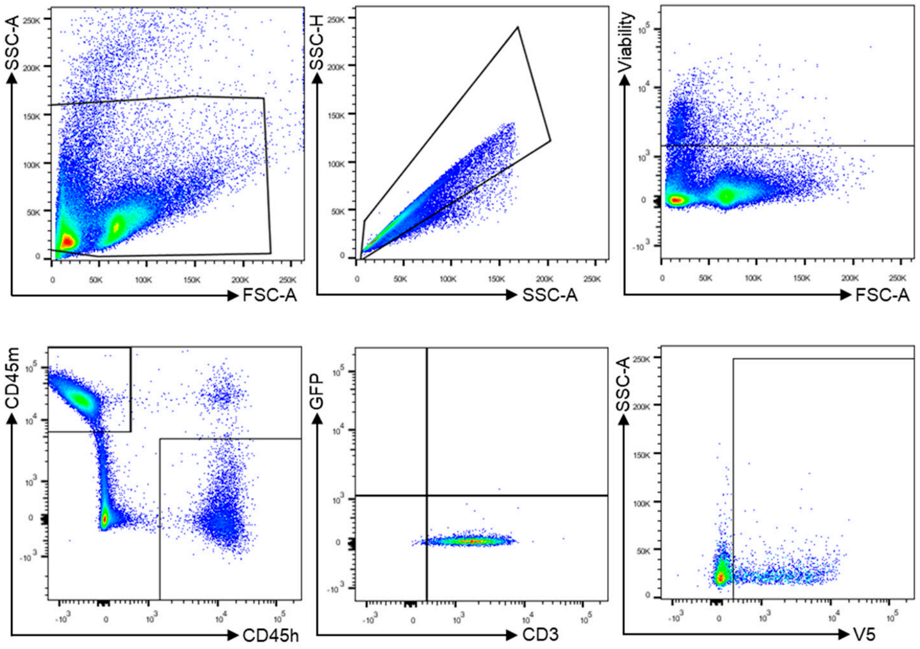

Figure S7 Gatting strategy of flow cytometry to select only alive human T or CAR-T cells by removing murine cells (CD45m-), and targeting V5 tag+ from CD45h+-CD3+ cells.

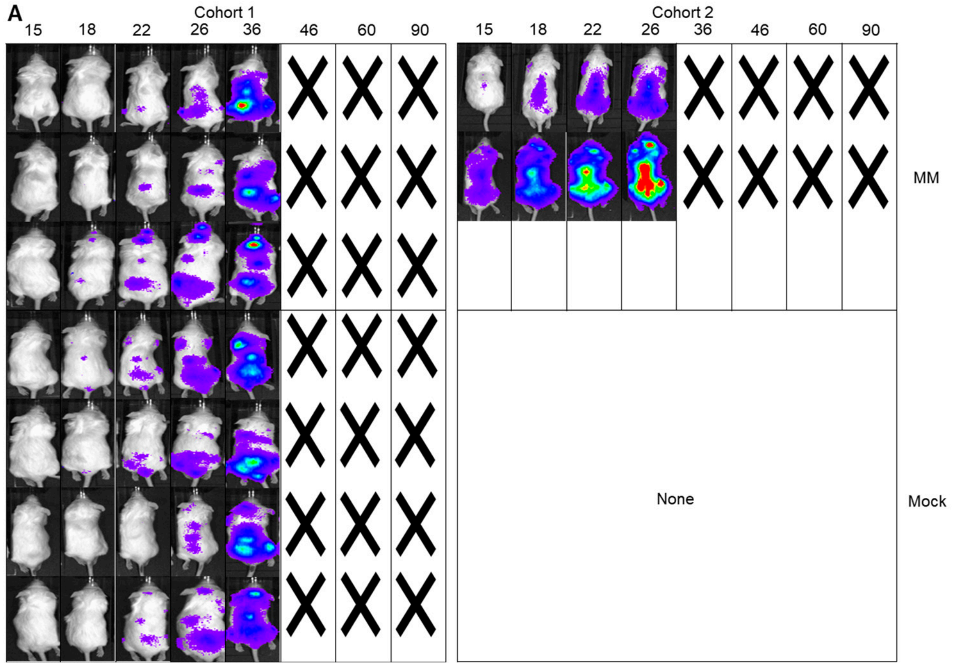

Figure S8 A. Representative bioluminescent images of mice from tumor only or Mock-T treatment groups at different timepoints.

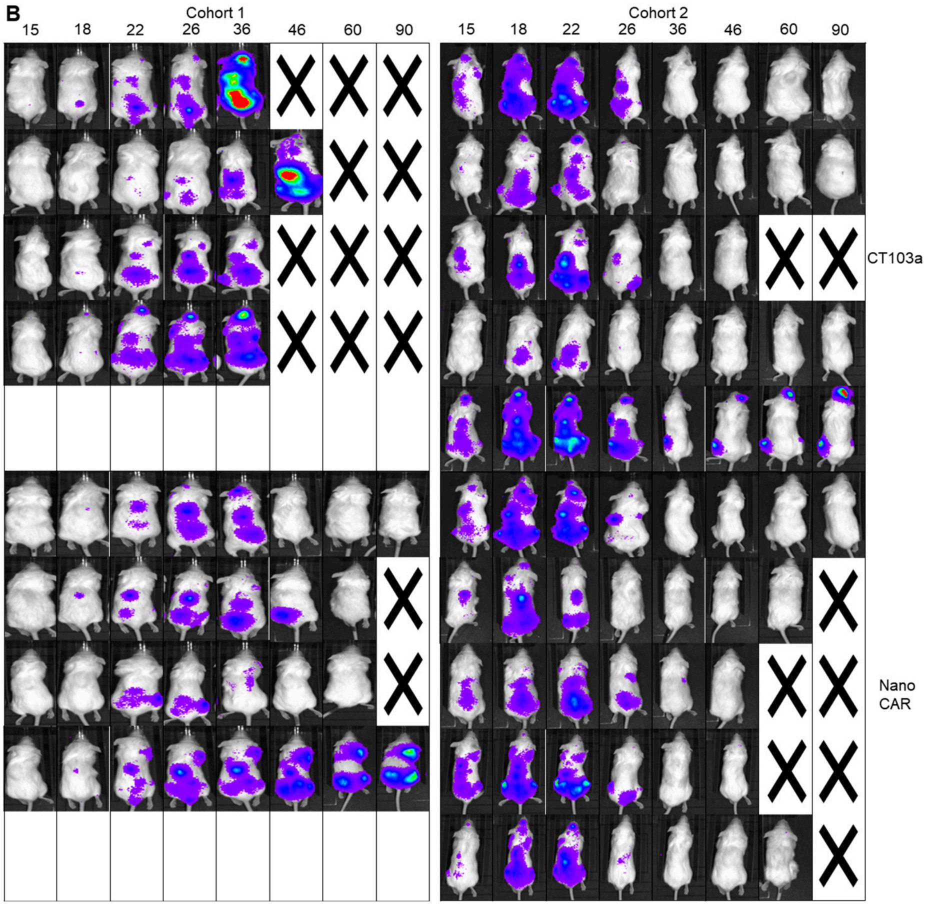

Figure S8 B. Representative bioluminescent images of mice from each CAR treatment group (CT103a, and nanoCAR) at different timepoints.

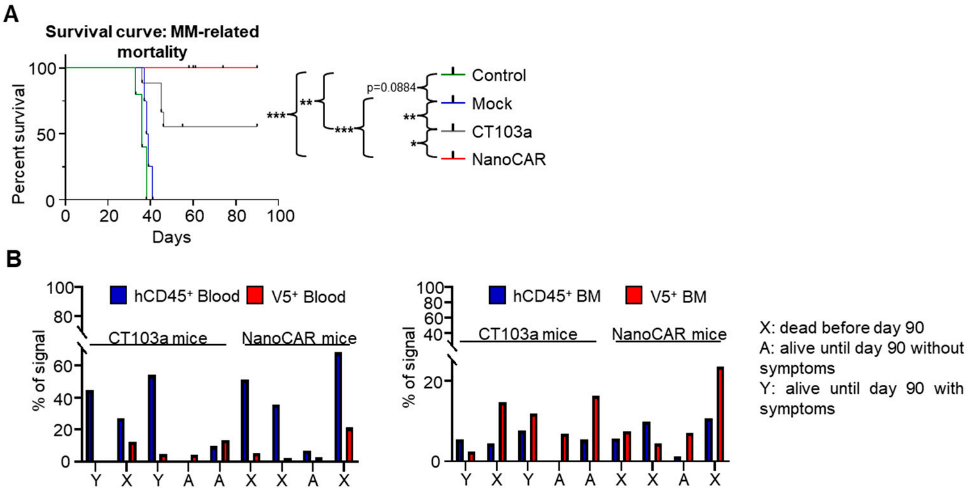

Figure S9 A. Kaplan-Meier survival curves of treated mice showing MM-related mortality for the in vivo experiments. **B.** Flow cytometry analysis of immune reconstitution in peripheral blood and BM of mice from the second cohort by analyzing frequency of human CD45+ (hCD45) cells and V5+ (CAR-T+) cells among hCD45+ populations. Mice in group A (sacrificed at day 90 without clinical symptoms) showed very low levels of circulating human $CD45^+$/CAR^+ cells, while retaining low but detectable levels of human $CD45^+$ and CAR^+ cells in blood, while higher levels of $CD45^+$ and CAR^+ cells were more detectable in the bone marrow. Mice in group Y (sacrificed at day 90 with clinical signs) displayed high levels of circulating human $CD45^+$ cells and very few or no circulating CAR^+ cells, while still retaining low residual human $CD45^+$ and CAR^+ cells in the bone marrow. The increase in circulating human $CD45^+$ cells in these mice is consistent with the development of

GvHD-like symptoms, which can occur in NSG mice infused with human T cells. Mice in group X (which died before day 90) also exhibited very high levels of human $CD45^+$ cells in blood and low levels of circulating CAR^+ cells. This profile is likewise compatible with GvHD-like disease. Notably, some mice in this group (one CT103A-treated and one NanoCAR-treated) retained substantial CAR^+ cell levels in the bone marrow, although blood CAR^+ levels were low.
